# *Acheta domesticus*: A Natural Source of Anti-Skin-Aging Ingredients for Cosmetic Applications

**DOI:** 10.3390/ph17030346

**Published:** 2024-03-07

**Authors:** Kankanit Yeerong, Panuwan Chantawannakul, Songyot Anuchapreeda, Thomas Rades, Anette Müllertz, Wantida Chaiyana

**Affiliations:** 1 Department of Pharmaceutical Sciences, Faculty of Pharmacy, Chiang Mai University, Chiang Mai 50200, Thailand; kankanit_yeerong@cmu.ac.th; 2 Bee Protection Laboratory, Department of Biology, Faculty of Science, Chiang Mai University, Chiang Mai 50200, Thailand; panuwan.c@cmu.ac.th; 3 Division of Clinical Microscopy, Department of Medical Technology, Faculty of Associated Medical Sciences, Chiang Mai University, Chiang Mai 50200, Thailand; songyot.anuch@cmu.ac.th; 4 Center of Excellence in Pharmaceutical Nanotechnology, Faculty of Pharmacy, Chiang Mai University, Chiang Mai 50200, Thailand; 5 Department of Pharmacy, Faculty of Health and Medical Sciences, University of Copenhagen, Universitetsparken 2, 2100 Copenhagen, Denmark; thomas.rades@sund.ku.dk (T.R.); anette.mullertz@sund.ku.dk (A.M.); 6 Bioneer:FARMA, Department of Pharmacy, University of Copenhagen, Universitetsparken 2, 2100 Copenhagen, Denmark; 7 Multidisciplinary and Interdisciplinary School, Chiang Mai University, Chiang Mai 50200, Thailand

**Keywords:** *Acheta domesticus*, anti-aging skincare, cosmetic, human dermal fibroblast, safety

## Abstract

*Acheta domesticus* is an edible insect, rich in nutritional value and considered a sustainable protein source. This study aimed to investigate the potential application of *A. domesticus* extracts for anti-skin-aging purposes. The extracts were prepared by maceration at ambient temperature with 95% ethanol or hexane and maceration in gentle heat (45 °C) with 95% *v*/*v* ethanol or DI water. The extracts were examined for total protein, phenolic, and flavonoid contents. Protein molecular weight distribution was analyzed. The safety of the extracts was investigated in terms of irritation and cytotoxicity. Biological activities relevant to the inhibition of skin aging were evaluated, including increasing transforming growth factor-beta 1 (TGF-β1) expression and inhibitory activities on collagenase and hyaluronidase. The aqueous extract from maceration in gentle heat had the highest total protein content (63 ± 1% *w*/*w*), total phenolic content (0.48 ± 0.03 mg GAE/g extract), TGF-β1 stimulating activities (33 ± 2 pg/mL), and collagenase inhibition (with a half maximal inhibitory concentration of 26 ± 1 µg/mL) among various extracts investigated. It caused no irritation to the hen’s egg chorioallantoic membrane and showed no cytotoxicity to human dermal fibroblasts and peripheral blood mononuclear cells. Therefore, aqueous *A. domesticus* extract is proposed as an innovative natural anti-skin-aging ingredient.

## 1. Introduction

Over 2000 insect species are used or investigated as food sources in 113 countries across Asia, Africa, Australia, and the Americas [1,2]. The Food and Agriculture Organization of the United Nations (FAO) recommends specific insect species as sustainable alternative protein and fat sources, resulting in increased attention also in Western nations as a potential resolution to malnutrition issues [3,4]. In Thailand, over 200 insect species have been consumed since ancient times, especially crickets, grasshoppers, mealworms, and giant water bugs [5]. As a result of the rising edible insect demand, insect farming has expanded around the world, and crickets are the most often farmed insect species. More than 20,000 cricket farms existed in Thailand in the year 2022 [6]. Various cricket species are farmed in Thailand, but the most common is *A. domesticus*, which is commonly known as house cricket.

*A. domesticus*, belonging to the Gryllidae family and a member of the Orthoptera order [7], originated in Southeast Asia but is now distributed throughout Europe, North Africa, Western Asia, the Indian Subcontinent, Australasia, Mexico, and North America [8]. *A. domesticus* has been accepted as a natural human food source due to its good texture, taste, and high protein content, ranging from 55 to 70% on a dry matter basis, which is higher than that found in plant-based protein sources and comparable to the content in animal-based protein sources [9]. Protein extracts from diverse sources have been suggested for use in cosmeceuticals. Extracts obtained from sardine and codfish have been noted for their antioxidant, anti-aging, and anti-hyperpigmentation properties [10]. Protein extracts from *Ulva intestinalis*, a green alga, have been documented to stimulate in vitro collagen and hyaluronic acid production by human dermal fibroblasts [11]. Additionally, egg yolk proteins have been identified for their antioxidant and immune-modulating properties [12]. *A. domesticus*, which has been reported to contain high protein content, would hence be appealing for cosmeceutical applications. The major amino acids of *A. domesticus* are glutamic acid, glutamine, lysine, valine, and arginine. Besides, *A. domesticus* has been reported to be a good source of lipids, which accounted for 19.32–24.91%, with the major fatty acids found being linoleic acid, palmitic acid, oleic acid, and stearic acid [13]. Furthermore, *A. domesticus* contains vitamins such as vitamin A, vitamin E, vitamin C, and vitamin B complex, as well as minerals such as potassium, calcium, iron, and magnesium [14,15].

Aside from their nutritional value, crickets have become increasingly interesting in recent years due to numerous biological activities, for instance, antioxidation, anti-obesity, anti-inflammatory, antimicrobial, antiangiogenic, antiproliferative, and anti-hypertensive [16,17,18,19,20]. Although *A. domesticus* is currently considered edible and is farmed for consumption as food, previous data regarding its biologically active compounds, which highlight it as a rich source of vitamins, fatty acids, and essential amino acids, make it likely to have the potential to be beneficial for topical applications as skin care. Furthermore, our previous study found that *A. domesticus* aqueous extract demonstrated the most potent biological activities, such as the inhibition of oxidation, matrix metalloproteinase (MMP), and elastase among several insect species [21]. Additionally, Di Mattia et al. (2019) also reported the highest antioxidant activity of the water-soluble extract from *A. domesticus* compared to other insect species [17]. Furthermore, the antioxidant capabilities of peptides are improved by heat treatment, which has a substantial influence and results in an increase in antioxidant peptide content [22]. Because of these biological properties, *A. domesticus* would be an appealing ingredient of anti-aging cosmeceutical products.

Apart from the potential of hydrophilic extract, the oil extracted from *A. domesticus* using hexane has also been reported to exhibit significantly high antioxidant activity [23]. The preceding data emphasized the prevalence of amino acids and fatty acids within *A. domesticus* and it revealed that both hydrophilic and lipophilic extracts derived from *A. domesticus* demonstrated noteworthy antioxidant potential. Nevertheless, there is a lack of previous data that definitively establish whether the insect’s potential primarily stems from its lipophilic or hydrophilic components. Conducting investigations to compare the biological activities of *A. domesticus* extracts obtained using solvents with different polarities would provide valuable guidance for future extract preparations.

The most important factor influencing the skin aging process is ultraviolet (UV) irradiation [24]. UV light can alter the homeostasis of the extracellular matrix (ECM) via mitogen-activated protein kinase (MAPK) cascades by inducing the expression of various types of MMPs, especially MMP-1 (collagenase-1), and suppress the generation of transforming growth factor beta 1 (TGF-β1), a cytokine that promotes collagen production in fibroblasts. Subsequently, ECM denaturation, wrinkle formation, and consequently premature skin aging occur [25,26]. Hence, anti-skin aging strategies have attempted to reduce ECM degradation and augment ECM synthesis [27].

Despite the extensive research regarding the nutritional aspect of *A. domesticus*, to our knowledge, information regarding the bioactive compounds and biological activities associated with the skin, specifically anti-skin-aging activity and safety, is sparse. Expanding the applications and utilization of crickets would increase their value. Due to the short production timeframe (approximately 45 days to reach market size) and the ease of the breeding process, enabling rapid and mass production of crickets [28], the concern about competition between food and non-food uses, previously experienced with biofuel sources, would be effectively addressed. Furthermore, cricket farming requires a relatively small land area and emits fewer greenhouse gases per unit of protein produced, making them an environmentally friendly protein source [29].

Therefore, the objective of this study was to explore the biological components, safety, and anti-skin-aging activities of *A. domesticus* extracts for further applications in the cosmetic/cosmeceutical area. As there were no previous data indicating whether the potential of the insect mainly came from lipophilic or hydrophilic components, the present study compared the biological activities of *A. domesticus* extracted using solvents with different polarities, which could serve as a guideline for further preparation of the extract.

## 2. Results and Discussions

### 2.1. Extracts of A. domesticus

**Figure 1:**
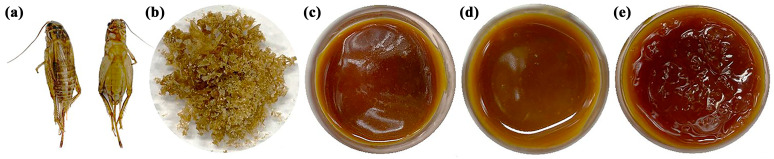
Physical appearance of *A. domesticus* (**a**), aqueous extract from thermal solvent extraction (AQ) (**b**), ethanolic extract from thermal solvent extraction (ED) (**c**), ethanolic extract from maceration (EM) (**d**), and hexane extract from maceration (HX) (**e**).

The physical appearance of *A. domesticus* is shown in Figure 1a. Prior to the extraction process, *A. domesticus* was dried according to the conditions suggested by Dobermann et al. (2019) [30]. The choice of the drying temperature of 45 °C for 36 h was based on their findings, which indicated that this condition resulted in the significantly highest percentage protein content in crickets (*Gryllus bimaculatus*) among various drying temperatures ranging from 32 °C to 120 °C [30]. The *A. domesticus* extracts were prepared in four different ways: by maceration with either deionized (DI) water or ethanol with the assistance of gentle heat (45 °C), a process also known as thermal solvent extraction [31], to reduce the extraction duration and prevent the extract from microbial growth and spoiling, and by maceration at ambient temperature with ethanol or hexane. Each *A. domesticus* extract had different physical characteristics as shown in Figure 1b–d. The aqueous extract (AQ) appeared as a dark brown solid powder, whereas the ethanolic extract from thermal solvent extraction (ED), ethanolic extract from maceration (EM), and hexane extract (HX) were paste-like brown-yellowish semisolid masses. The brown color of the extract was attributed to melanin, a major insect pigment [32]. Notably, *A. domesticus* extracts from the thermal solvent extraction (performed at 45 °C) had a darker brown color than the extracts from maceration, which was performed at ambient temperature (~30 °C). The likely explanation for this color difference could be non-enzymatic browning, i.e., the Maillard reaction, of protein-rich compounds, which occurs under thermal processes and depends on storage conditions [33]. Moreover, each extract had a characteristic odor. AQ had a food-like smell, with hints of salt and seafood, whereas ED and EM had an essence of acid. In contrast, the odor of HX was like oil with some rancidity. Grossmann et al. (2021) reported that 2-/3-methylbutanoic acid, phenylacetaldehyde, 2,3-butanedione, 2-acetyl-1-pyrroline, furaneol, trimethylpyrazine, and *p*-cresol were identified as odor-active compounds of *A. domesticus* protein, protein hydrolysates, and their Maillard products [34]. Additionally, Spano et al. (2023) demonstrated the volatile profiles of *A. domesticus* powder, with hexadecanoic acid (54.6 ± 0.02%) being the most abundant compound, followed by *p*-cymene (17.2 ± 0.02%), *γ*-terpinene (16.8 ± 0.03%), *β*-myrcene (3.5 ± 0.08%), 6-ethyl-2-methyl-decane (2.5 ± 0.03%), 1,2-dipropenyl-cyclobutane (1.5 ± 0.02%), 4-carene (1.5 ± 0.02%), *α*-thujene (0.9 ± 0.02%), linalyl butyrate (0.8 ± 0.02%), and *β*-thujene (0.7 ± 0.03%), respectively [35].

**Figure 2:**
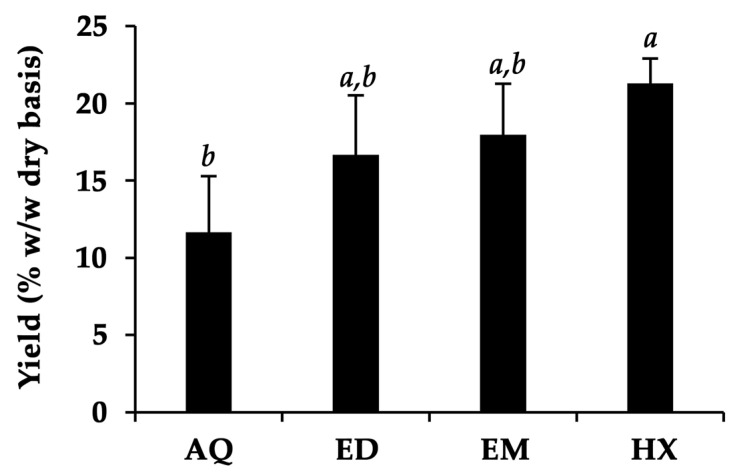
The yields of *A. domesticus* extracts, including aqueous extract from thermal solvent extraction (AQ), ethanolic extract from thermal solvent extraction (ED), ethanolic extract from maceration (EM), and hexane extract from maceration (HX). The data are shown as mean ± SD (*n* = 3). The lower-case letters (*a* and *b*) indicate significant differences between the yields of the respective *A. domesticus* extracts. Statistical significance was analyzed using one-way ANOVA followed by a post hoc Tukey test (*p* < 0.05).

The yields of *A. domesticus* extracts are shown in Figure 2. Despite the fact that other studies found that thermal solvent extraction had a better extraction efficiency than conventional maceration due to the higher extraction temperature [36,37], the current investigation found the opposite outcome. HX and EM, prepared by maceration for three cycles of 24 h, tended to yield higher extract amounts than AQ and ED, prepared by thermal solvent extraction for 3 h. The likely explanation for this finding could be the longer duration of extraction and the number of extraction cycles used in the maceration [38,39].

On the other hand, HX was found to have the highest yield, accounting for more than 20% *w*/*w* on a dry basis. This is likely due to the efficiency of hexane in extracting lipids from *A. domesticus*, despite its lower content of fat than protein [9,40]. The findings were consistent with a related study by Ndiritu et al. (2017), who reported that the yield of an *A. domesticus* hexane extract was higher than that of an aqueous extract [32]. The aqueous and ethanol, which should effectively extract the polar components according to the “like-dissolves-like” rule, yielded lower extract contents than hexane. The most plausible explanation for this finding could be that maceration, a traditional and less complex procedure based on the principle of diffusion (Fick’s law), is not always applicable and especially efficient for extracting these polar fractions, despite heating the system, which could increase the speed of maceration [41]. Nonetheless, the thermal solvent extraction process, particularly using water (“green extraction”), provides a more cost-effective, safe, short-duration, and environmentally sustainable alternative to maceration.

### 2.2. Chemical Composition of A. domesticus Extracts

The chemical composition of *A. domesticus* extracts is shown in Table 1. Protein, alongside phenolic and flavonoid components, was detected in *A. domesticus* extracted using various solvents. *A. domesticus* has been reported to contain a higher protein content (72.0%) than other cricket species, including *Gryllus bimaculatus* De Geer (60.7%), *Scapsipedus icipe* (56.8%), and *Gryllodes sigillatus* (70.0%) [42,43]. In the present study, AQ contained the significantly highest protein concentration of 63 ± 1% *w*/*w* dry weight basis (*p* < 0.05). These results comply with previous studies stating that the protein content range of adult *A. domesticus* was from 56.2% to 72.0% depending on the source, feed composition, origin, sex, rearing ecology, stage of life, etc. [44,45,46,47]. The present study suggested water as the most suitable solvent for protein isolation, followed by ethanol and hexane, respectively (*p* < 0.05). Since most proteins are water-soluble due to the polar amino acids in the molecules, e.g., glutamic acid, aspartic acid, and serine [48], proteins could be extracted well using a solvent with high polarity (DI water) compared to a semi-polar solvent (ethanol) and a non-polar solvent (hexane). In addition, several proteins can be denatured or precipitated by organic solvents [49]. This might be another justification for the lower protein concentration of *A. domesticus* extracts from ethanol and hexane extraction, in addition to the well-known solubility of most proteins in aqueous solutions. A previous study reported the presence of nineteen amino acids and their derivatives, along with five organic acids (acetic, formic, fumaric, lactic, and succinic acids), as well as additional compounds such as choline and glycerol, in the hydroalcoholic extract of *A. domesticus* [35]. On the other hand, the hexane extract was found to mostly contain fatty acids, with linoleic acid (38.1%) being the most abundant, followed by palmitic acid (27.8%), oleic acid (21.0%), stearic acid (10.4%), linolenic acid (2.5%), and pentanoic acid (0.2%) [35]. In addition, terpenes and methyl-branched hydrocarbons were detected in the hexane extracts [35].

Phenols and flavonoids are usually present in small amounts in natural extracts, particularly when derived from insects, because they are considered minor chemical constituents [50]. However, significant differences were observed in the *A. domesticus* extracts from the various solvents. The present study revealed that AQ contained the significantly highest phenolic content, whereas EM contained the highest total flavonoid content (*p* < 0.05). The explanation for this is likely due to the compatibility of the extracting solvent with the extracting compounds. As most phenolic compounds are hydrophilic molecules, they can be extracted using polar solvents, particularly water (ε = 78.4) [51,52]. Flavonoids, on the other hand, are slightly hydrophobic molecules that prefer semi-polar solvents such as ethanol (ε = 25.4) [53]. The phenolic contents found in this study were in good agreement with the work of Del Hierro et al. (2020), which had reported that the highest total phenolic content was detected using a 1:1 *v*/*v* ethanol–water extract of *A. domesticus* when compared to an ethanolic extract [54].

### 2.3. Protein Molecular Weight Distribution of A. domesticus Extracts

**Figure 3:**
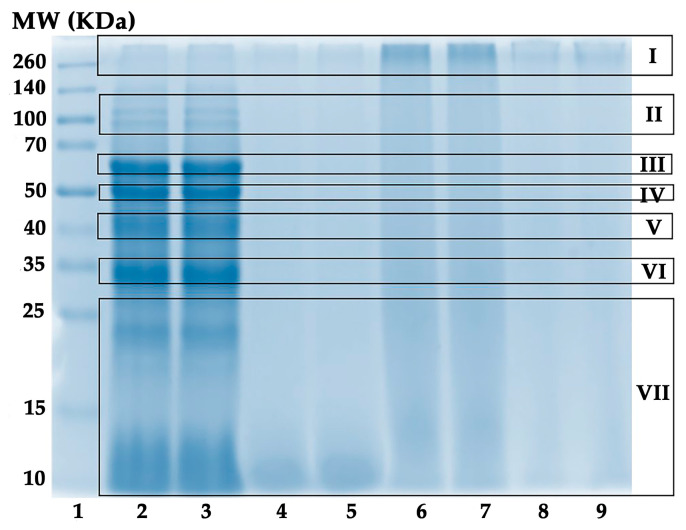
Protein molecular weight distributions. Lane 1: protein molecular weight standard; lanes 2 and 3: *A. domesticus* aqueous extract from thermal solvent extraction (AQ); lanes 4 and 5: *A. domesticus* ethanolic extract from thermal solvent extraction (ED); lanes 6 and 7: *A. domesticus* ethanolic extract from maceration (EM); and lanes 8 and 9: *A. domesticus* hexane extract from maceration (HX).

Since protein was identified in all *A. domesticus* extracts, the molecular weight distribution of protein in each *A. domesticus* extract was examined. The results, as shown in Figure 3, indicated that except for AQ (lanes 2 and 3), which showed a variety of protein bands, none of the *A. domesticus* extracts exhibited a clear and intense protein band due to the insolubility of the extract in buffer, protein degradation, denaturation, or precipitation [47].

The protein bands of AQ were found in the molecular weight (MW) range from 10 to 260 kDa. The possible protein types for each protein band are listed in Table 2. The most intense protein bands of AQ were found at molecular weights of around 60 kDa (III), 50 kDa (IV), 40 kDa (V), and 33 kDa (VI). The band around 60 kDa (III) could be β-glycosidases, digestive enzymes found in insect digestive systems that hydrolytically catalyze the β-glycosidic linkage of glycosides [55,56]. On the other hand, AQ was found to contain myofibrillar proteins, including tubulin (50 kDa), troponin T (46 kDa), actin (42 kDa), and tropomyosin (33 kDa) [57]. Aside from actin, the protein band detected around 40 kDa (V) could be monomeric arginine kinase (41 kDa), an ATP phosphotransferase found in invertebrates that catalyzes arginine phosphorylation [58,59,60]. Since most myofibrillar proteins, which are embedded in the exoskeleton and play a significant functional role in insect muscle contraction, have been reported to be thermostable [57], they could be found in AQ (extracted by thermal solvent extraction using gentle heat). The results are in line with previous studies, which found myofibrillar proteins in *A. domesticus* [57]. In addition, Montowska (2019) detected abundant myofibrillar protein in *A. domesticus* powder, i.e., myosin heavy chain, actin, tropomyosin, tubulin, paramyosin, and troponin T [57].

Additionally, two faint protein bands were detected around 100 kDa (II). They could be sarcoplasmic/endoplasmic reticulum calcium ATPase or SERCA (111 kDa) and alpha-actinin (106 kDa), which act as key regulators of muscle contraction in insects [61,62]. SERCA is an enzyme that transports calcium ions to the sarcoplasmic reticulum, causing muscular relaxation [63], whereas alpha-actinin is a cytoskeletal protein that binds to actin filaments to stabilize the muscle contractile apparatus and aids in force transmission across the muscle fibers [64]. Our findings align with a related study reporting that SERCA and alpha-actinin were identified in *A. domesticus* powders by UHPLC-QTOF-MS/MS analysis [57].

In addition, some protein bands with MW ranging between 10 and 25 kDa (VII) were observed in AQ. The band between 15 and 25 kDa could be cuticle proteins or the product of proteolytic degradation, since the findings agreed with a related study reporting cuticle protein bands (14–32 kDa) found in cricket powder [42,55].

In contrast to AQ, the accumulation of protein on the top of the lanes with MW > 260 kDa (I) could be a result of protein glycation, cross-linking, or aggregation [57,65], intensely in EM (lanes 6 and 7) and faintly in HX (lane 8 and 9). A possible explanation could be attributed to the difference in the extraction techniques. Generally, a higher temperature can disrupt protein structures, resulting in protein unfolding and denaturation [66]. For EM and HX, which were prepared by maceration at ambient temperature, their large proteins were obstructed and could not migrate through the gel.

Nonetheless, data on *A. domesticus* proteins in the NCBI BLAST and UniProtKB databases are still lacking [57,67], so many *A. domesticus* proteins have not yet been identified and sequenced.

### 2.4. Irritation Properties of A. domesticus Extracts

**Figure 4:**
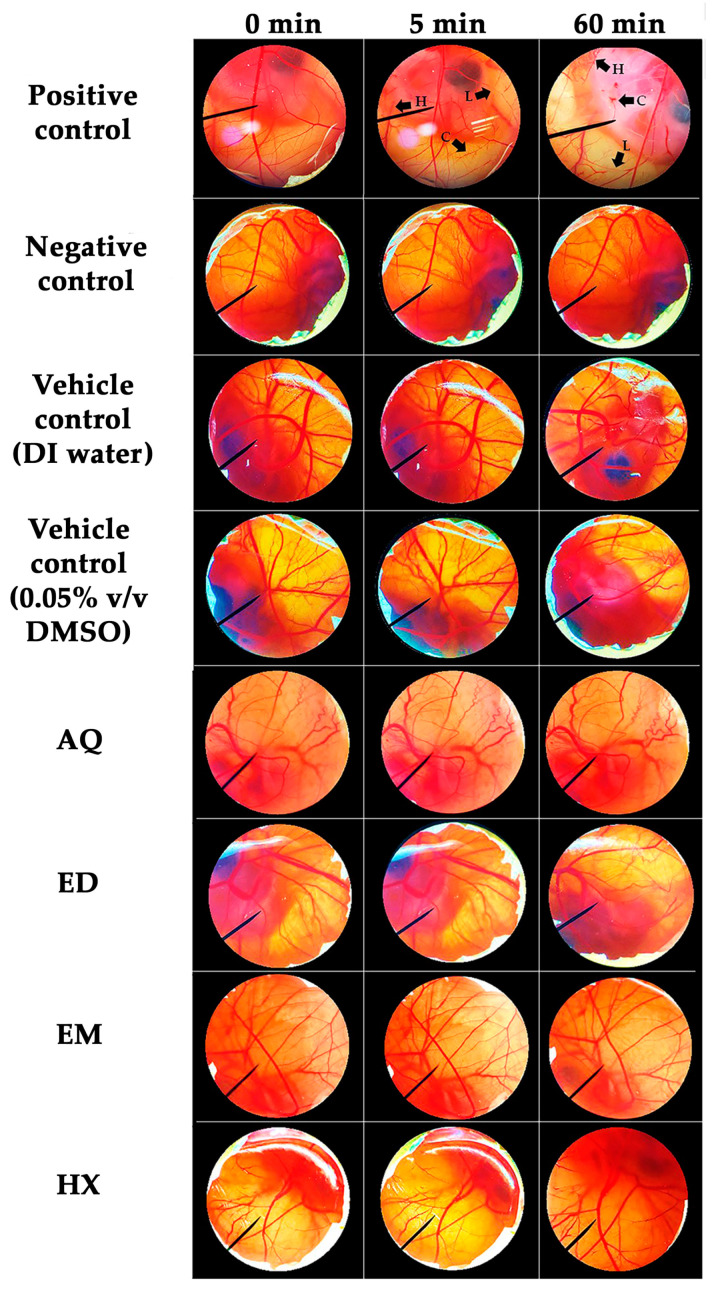
Effects of positive control (1% *w*/*v* SLS), negative control (0.9% *w*/*v* NaCl solution), vehicle controls (DI water and 0.05% *v*/*v* DMSO), and *A. domesticus* extracts, including aqueous extract from thermal solvent extraction (AQ), ethanolic extract from thermal solvent extraction (ED), ethanolic extract from maceration (EM), and hexane extract from maceration (HX), on the CAM with contact times of 0, 5, and 60 min. The black arrows indicate damaged sites on the CAM after application. L represents vascular lysis, H represents hemorrhage, and C represents coagulation.

The hen’s egg test-chorioallantoic membrane (HET-CAM) test is an alternative method to examine the irritation potential of test compounds that could substitute the Draize rabbit eye test [68]. Therefore, it was used to investigate the safety of *A. domesticus* extracts. The effects of *A. domesticus* extracts on the CAM are shown in Figure 4. The positive control (1% *w*/*v* SLS) immediately exposed strongly irritative signs, including hemorrhage, coagulation, and vascular lysis on the CAM after application, which became more pronounced after 60 min with an irritation score (IS) of 12  ±  1, classified as strongly irritant. In contrast, the negative control (0.9% *w*/*v* NaCl solution) and vehicle controls (DI water and 0.05% *v*/*v* DMSO) revealed no irritation (IS = 0). No signs of irritation were detected on the CAM during 60 min of *A. domesticus* extract application. These findings confirm the safety of all the *A. domesticus* extracts for topical applications. The results were in line with our previous study, which reported no skin irritation of *A. domesticus* extracts in both in vitro HET-CAM and in vivo human patch tests in volunteers [21]. In addition, no allergens have been reported within crickets, and specific food-related allergies resulting from *A. domesticus* consumption have not been documented in Europe or regions where cricket consumption is prevalent [69].

### 2.5. Cytotoxic Effects of A. domesticus Extracts

*A. domesticus* extracts were assessed for cytotoxicity on human dermal fibroblasts (Hs68 cells) and peripheral blood mononuclear cells (PBMCs). Hs68 cells are a major cell type predominant in the dermis. Since the intention of *A. domesticus* extract application is as an active cosmetic ingredient, which is applied topically to the skin, Hs68 cells were used in the present study. Although the entire *A. domesticus* extract is typically not expected to easily penetrate the intact skin, some specific compounds within the extract may indeed have the capacity to penetrate the skin barrier and be absorbed into the blood circulation, resulting in systemic effects. However, the cytotoxicity of *A. domesticus* extract on PBMCs, the major cells in the human body’s immune system and usually used as a representative cell type in toxicology studies [70,71], was studied to ensure that it would not have any toxic or negative consequences even if absorbed into the blood circulation.

**Figure 5:**
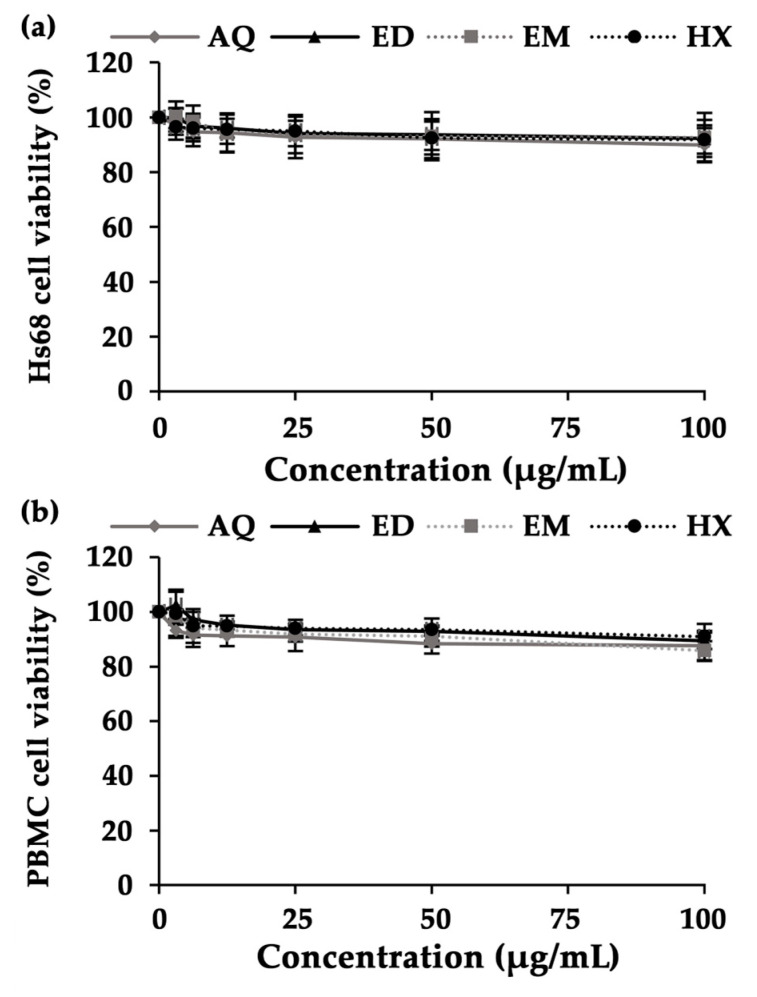
Cytotoxic effects of *A. domesticus* extracts on Hs68 cells (**a**) and PBMCs (**b**) after 48 h of treatment. *A. domesticus* extracts comprised aqueous extract from thermal solvent extraction (AQ), ethanolic extract from thermal solvent extraction (ED), ethanolic extract from maceration (EM), and hexane extract from maceration (HX). The data are shown as mean ± SD (*n* = 3).

The cytotoxicity effects of *A. domesticus* extracts are presented as dose–response cell viability curves in Figure 5. Remarkably, none of the *A. domesticus* extracts showed cytotoxic effects on Hs68 cells or PBMCs. The Hs68 and PBMC cell viabilities were higher than 80%, even at the maximum tested concentration of 100 µg/mL. Therefore, it can be stated that all *A. domesticus* extracts are safe and show no cytotoxicity.

### 2.6. TGF-β1-Stimulating Activities of A. domesticus Extracts

As collagen is the most prevalent component of the extracellular matrix (ECM) in the dermis layer and is responsible for skin integrity, the stimulation of collagen production would be beneficial for anti-skin-aging purposes [72]. Transforming growth factor beta-1 (TGF-β1), a polypeptide cytokine belonging to the TGF-β superfamily, plays an essential role in ECM formation and fibroblast function regulation, i.e., differentiation and proliferation [73,74]. The TGF-β/Smad signaling pathway directly activates many ECM genes and promotes the expression of specific ECM components, including collagens, fibronectins, and decorins [75].

**Figure 6:**
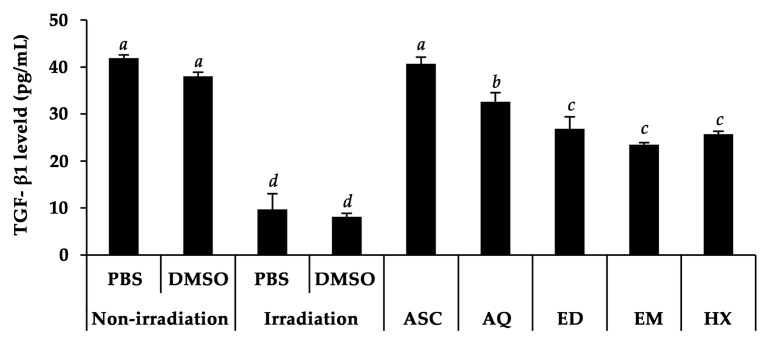
Levels of TGF-β1 stimulated by *A. domesticus* extracts, including aqueous extract from thermal solvent extraction (AQ), ethanolic extract from thermal solvent extraction (ED), ethanolic extract from maceration (EM), and hexane extract from maceration (HX) at a concentration of 100 µg/mL in Hs68 cells after UVB exposure. Ascorbic acid (ASC) was used as a positive control. PBS and DMSO were used as vehicle controls. The data are shown as mean ± SD (*n* = 3). The lower-case letters (*a*, *b*, *c*, and *d*) indicate significant variability among *A. domesticus* extracts. Statistical significance was analyzed using one-way ANOVA followed by a post hoc Tukey test (*p* < 0.05).

The effects of *A. domesticus* extracts on TGF-β1 expression in Hs68 cells are shown in Figure 6. Through the downregulation of TGF-β type II receptor (TβRII) mRNA and protein, UV irradiation reduced cellular responsiveness to TGF-β, which lowered the synthesis of TGF-β1 [76]. The reduction of TGF-β1 expression can induce MMP activities, particularly MMP-1 and -3, and subsequently reduce the synthesis of pro-collagen type I, which results in skin aging [77]. A recent study revealed that the TGF-β1 level on Hs68 cells significantly declined after UVB exposure when compared to non-irradiated cells. Interestingly, *A. domesticus* extracts could prevent the suppression of TGF-β1. Among the various *A. domesticus* extracts, AQ treatment resulted in the significantly highest TGF-β1 level in Hs68 cells after UVB exposure (*p* < 0.05). These results are in line with a previous study showing that black cricket extract was able to raise the TGF-β mRNA level in HR-1 hairless mice after UVB radiation [78]. Furthermore, Jeong et al. (2020) discovered that the methanol extract of *G. bimaculatus* (black cricket) could mitigate UVB-induced collagen degradation in Hs68 fibroblast cells [79]. Consequently, *A. domesticus* possessed the potential to delay collagen degradation through the impact of its composition.

### 2.7. Anti-Skin-Aging Activities of A. domesticus Extracts

Skin aging can be caused by various mechanisms, and the most crucial factor is the degradation of ECM, including collagen and elastin fibers [80]. Matrix metalloproteinase-1 (MMP-1), commonly known as collagenase, is a zinc-dependent endopeptidase involved in ECM deterioration resulting in coarseness, deep wrinkles, and inflexible skin [81]. Additionally, aging skin is less hydrated, and wrinkles are more visible. The primary molecule responsible for skin moisture is hyaluronic acid (HA), a non-sulfated glycosaminoglycan that can bind and hold water molecules [82]. Consequently, an imbalance between HA production and degradation leads directly to skin aging [83].

**Figure 7:**
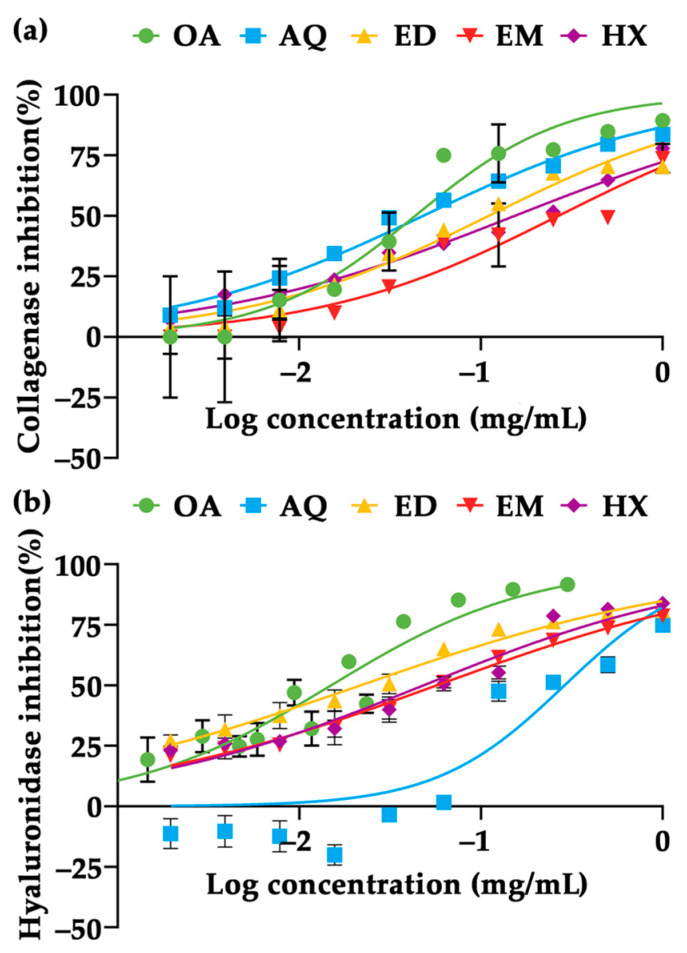
Dose–response curves of collagenase inhibition (**a**) and hyaluronidase inhibition (**b**) of *A. domesticus* extracts, including aqueous extract from thermal solvent extraction (AQ), ethanolic extract from thermal solvent extraction (ED), ethanolic extract from maceration (EM), and hexane extract from maceration (HX). Oleanolic acid (OA) was used as a positive control. The data are expressed as mean ± SD (*n* = 3).

In the present study, the collagenase and hyaluronidase inhibitory effects of *A. domesticus* extracts were investigated and the dose–response curves are shown in Figure 7. The calculated 50% inhibitory concentration (IC_50_) of each extract is also shown in Table 3. AQ outperformed the other *A. domesticus* extracts in the present study in collagenase inhibition, whereas ED exhibited the most significant hyaluronidase inhibition, comparable to that of oleanolic acid. The results are in line with a previous study reporting that the ethanolic extract of *G. bimaculatus*, known as black cricket, enhanced hyaluronic acid content and reduced transepidermal water loss (TEWL) on HR-1 hairless male mice [78].

**Figure 8:**
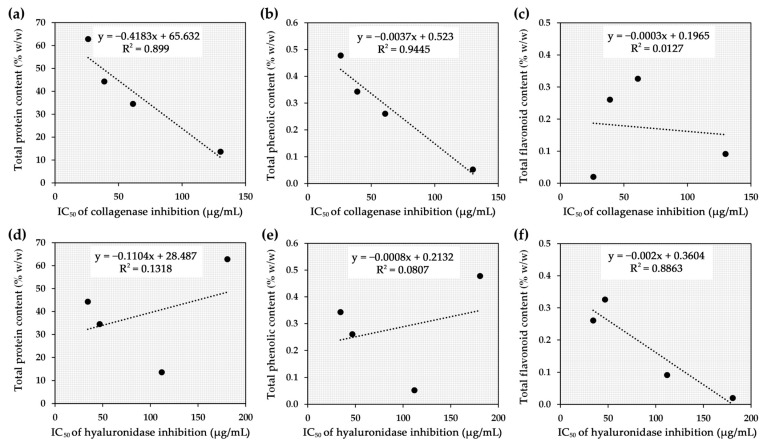
Correlation between half-maximal inhibitory concentration (IC_50_) against collagenase and total protein content (**a**), total phenolic content (**b**), and total flavonoid content (**c**), along with correlation between IC_50_ against hyaluronidase and total protein content (**d**), total phenolic content (**e**), and total flavonoid content (**f**).

The correlations between the bioactive components of *A. domesticus* extracts and their biological activities are shown in Figure 8. Proteins and phenolic compounds exhibited a consistent trend as essential components responsible for collagenase inhibitory abilities in *A. domesticus* extracts, demonstrating strong correlations with R^2^ values of 0.899 and 0.9945, respectively. In contrast, flavonoids were found not to be related to collagenase inhibition (R^2^ = 0.0127). The findings in the current study were consistent with a previous study that found walnut protein hydrolysates significantly inhibited MMP-1 activity [84]. Moreover, cod skin gelatin hydrolysates were found to have a strong inhibitory effect on MMP-1 expression [85]. However, an obvious difference was found in the hyaluronidase inhibition. While proteins and phenolic compounds displayed a low correlation with hyaluronidase inhibition (R^2^ = 0.1318 and 0.0807, respectively), flavonoids exhibited a strong correlation with an R^2^ value of 0.8863. These findings implied that flavonoids in *A. domesticus* were responsible for their potential to inhibit hyaluronidase activity. Flavonoids have been reported to inhibit hyaluronidase activity by binding to its specific site through electrostatic and hydrophobic interactions, altering the microenvironment of the hyaluronidase enzyme and thereby reducing its activity [86]. Therefore, it could be inferred that proteins and phenolic compounds are essential components responsible for collagenase inhibitory ability in *A. domesticus* extracts but not for hyaluronidase inhibition. On the contrary, flavonoids were suggested to be essential for anti-hyaluronidase. Therefore, *A. domesticus* has great potential to be used as a bioactive cosmeceutical ingredient. Its potential as an appealing source of bioactive cosmeceutical ingredients lies in its capacity to breed in a brief 45-day timeframe, the ease of the breeding process, its suitability for small-scale production, minimal investment needs, and the absence of advanced technology prerequisites [87]. It could enhance the agricultural economic prospects of cricket farming and create a valuable opportunity to generate bioactive compounds. Nowadays, people engage in cricket farming due to a combination of economic reasons, ease of operation, affordability, and perceived market advantages [88]. Low investment and rapid payback provide economic advantages of the potential *A. domesticus* production [28]. The production quantity should be adequate for the cosmetic/cosmeceutical industry, since it utilizes minimal space, making it easily expandable, and allows for quick production. Moreover, considering its efficacy as a potent anti-aging ingredient, it does not necessitate significant quantities for the formulation. Additional research into the amino acid sequences of the proteins and protein hydrolysates is proposed for in-depth comprehension and might be valuable for the quality control of *A. domesticus* extracts when used as an active component in anti-aging cosmeceutical products.

## 3. Materials and Methods

### 3.1. Insect Material

Frozen *A. domesticus* with the initial weight of 10 kg was acquired from the Ruamchok market in Chiang Mai, Thailand. The frozen *A. domesticus* was thawed at ambient temperature and dried using a hot-air oven at 45 °C (Memmert, Schwabach, Germany) for 36 h until a consistent weight was obtained [30]. Subsequently, dried *A. domesticus* was converted into a fine powder using a Moulinex™ blender, model number LM2070BD (Moulinex SA, Bagnolet, France), and stored in a sealed container at −20 °C for further analysis.

### 3.2. Chemical Reagents

Ficoll-Paque reagent, 3-(4,5-dimethylthiazol-2-yl)-2,5-diphenyltetrazolium bromide (MTT), quercetin, gallic acid, collagenase from *Clostridium histolyticum*, N-[3-(2-furyl)acryloyl]-Leu-Gly-Pro-Ala (FALGPA), hyaluronidase from bovine testes, and hyaluronic acid sodium salt from *Streptococcus equi* were purchased from Sigma Aldrich (St. Louis, MO, USA). Disodium hydrogen phosphate (Na_2_HPO_4_), potassium dihydrogen phosphate (KH_2_PO_4_), potassium chloride (KCl), sodium carbonate (Na_2_CO_3_), and sodium chloride (NaCl) were purchased from Thermo Fisher Scientific (Waltham, MA, USA). Bovine serum albumin (BSA) and Bradford reagent were purchased from Merck (Darmstadt, Germany). Sodium hydroxide (NaOH), dimethyl sulfoxide (DMSO), hydrochloric acid (HCl), and ethanol (all analytical grade) were purchased from RCI Labscan Co., Ltd. (Bangkok, Thailand). RPMI-1640 medium, Dulbecco’s modified Eagle’s medium (DMEM), L-glutamine, and penicillin-streptomycin were purchased from GIBCO Invitrogen (Grand Island, NY, USA). Fetal bovine serum (FBS) was obtained from Biochrom AG (Berlin, Germany).

### 3.3. Preparation of A. domesticus Extracts

*A. domesticus* fine powder was subjected to solvent extraction, both with and without thermal processing. In the case of extraction without thermal processing, *A. domesticus* fine powder was separately macerated in either 95% ethanol or hexane in well-closed glassware for 3 cycles, with each cycle lasting 24 h at ambient temperature. For the thermal solvent extraction, *A. domesticus* fine powder was separately subjected to thermal solvent extraction with either distilled water or 95% ethanol for 3 h at 45 °C using a water bath (Memmert, Schwabach, Germany). Following both extraction methods, the supernatants were collected, filtered using Whatman No. 1 filter papers, and then subjected to centrifugation at 3000× *g* using a laboratory centrifuge (model number MPW-352R, MPW, Warsaw, Poland). Subsequently, the supernatants were subjected to drying either using a rotary evaporator (Eyela, Tokyo, Japan) or a freeze dryer (model number Beta 2–8 LD-plus, Christ, Osterode am Harz, Germany), resulting in four distinct extracts: EM, HX, AQ, and ED. Each extract was characterized for its external appearance, in terms of general appearance, color, and odor. All extracts were stored in sealed containers at –20 °C for further analysis.

### 3.4. Chemical Composition Analysis of A. domesticus Extracts

#### 3.4.1. Total Protein Content Determination

*A. domesticus* extracts were examined for total protein content using the Bradford assay, following a previous study [89]. The standard curve was created using BSA (concentrations ranging from 50–2000 µg/mL). The results are shown as the percentage of total protein content compared to the weight of the *A. domesticus* extract. Three experiments were performed independently.

#### 3.4.2. Total Phenolic Content Determination

The *A. domesticus* extracts were examined for total phenolic content using the Folin–Ciocalteu assay in accordance with Jiamphun and Chaiyana [90]. The standard curve was established using gallic acid (concentrations ranging from 0.25 to 100 µg/mL). The results were reported as milligrams of gallic acid equivalent (GAE) per gram of *A. domesticus* extract. Three experiments were performed independently.

#### 3.4.3. Total Flavonoid Content Determination

*A. domesticus* extracts were examined for total flavonoid content using the aluminium chloride (AlCl_3_) colorimetric method with minor modifications [91]. The standard curve was constructed using quercetin (concentrations ranging from 0.25 to 100 µg/mL). The results were reported as milligrams of quercetin equivalent (QE) per gram of *A. domesticus* extract. Three experiments were performed independently.

### 3.5. Protein Molecular Weight Distribution Analysis

The *A. domesticus* extracts were inspected for molecular weight distribution using sodium dodecyl sulfate-polyacrylamide gel electrophoresis (SDS-PAGE) adapted from Montowska et al. [57]. In brief, 10 μL of each *A. domesticus* extract (10 mg/mL) was mixed with 10 μL of Laemmli sample buffer (Bio-Rad, Richmond, CA, USA) and heated at 90 °C for 5 min. The mixtures were loaded into each well of 12% SDS-PAGE gel. The gel was subjected to the electrophoresis cell with a constant current of 100 V. Thermo Scientific™ Spectra™ Multicolor Broad Range Protein Ladder (Thermo Fisher Scientific (Waltham, MA, USA) was utilized as a molecular weight protein standard. After that, the gel was washed using 10% *v*/*v* acetic acid with 40% *v*/*v* methanol and stained with 0.025% *w*/*v* Coomassie Brilliant Blue R-250 (Bio-Rad, Richmond, CA, USA) for 1 h. Then, the gels were de-stained using 10% *v*/*v* acetic acid with continuous agitation until the background was clear. The SDS-PAGE gel image was captured, and protein bands were analyzed for their molecular weight using a gel documentation system, model number Gel Doc^TM^ EZ Imager (Bio-Rad, Richmond, CA, USA). Two experiments were performed independently.

### 3.6. Determination of Irritation Properties of A. domesticus Extracts

The *A. domesticus* extracts were investigated for their irritation potentials using the HET-CAM method adapted from Somwongin et al. [92]. The HET-CAM assay is a substitutive method for the prediction of the local irritative potentials of test substances on the CAM of a hen’s egg by observing the irritation signs that occur in the blood vessels [93]. The irritation properties were described as irritation score (IS) and calculated using the following equation [94]:

IS = [((301 − *H*))/300 × 5] + [((301 − *L*))/300 × 7] + [((301 − *C*))/300 × 9, (1)

where *H* is the onset of vascular hemorrhage occurring, *L* is the onset of vascular lysis occurring, and *C* is the onset of vascular coagulation occurring on the CAM. The IS was classified as follows: 0.0–0.9 as non-irritating, 1.0–4.9 as slightly irritative, 5.0–8.9 as moderately irritative, and 9–21 as strongly irritative. A solution of 1% *w*/*v* SLS was used as a positive control and 0.9% *w*/*v* NaCl solution was used as a negative control. Two experiments were performed independently.

### 3.7. Determination of Cytotoxicity of A. domesticus Extracts

The *A. domesticus* extracts were investigated for cytotoxicity using a 3-(4,5-dimethylthiazol-2-yl)-2,5-diphenyltetrazolium bromide (MTT) assay following Viriyaadhammaa et al. [95] on Hs68 dermal fibroblast cells (Japanese Collection of Research Bioresources Cell Bank, Osaka, Japan) and PBMCs. The cytotoxicity study on PBMCs was permitted by the Human Research Ethics Committee, Faculty of Associated Medical Sciences, Chiang Mai University (ethical number AMSEC-64EX-0.29). The clinical trial was registered and accepted by the Thai Clinical Trials Registry (TCTR) Committee on 9 August 2022. The accessible TCTR identification number is TCTR20220809007 and the valid URL of the registry is https://www.thaiclinicaltrials.org/export/pdf/TCTR20220809007.

PBMCs were collected from five blood donors. The blood was diluted with the same volume of PBS. Consequently, Ficoll–Paque reagent was added to the diluted blood and then centrifuged at 400× *g* for 30 min, resulting in several layers of blood separation. The PBMC layer was collected and washed 3 times with PBS and cultured in RPMI-1640 media supplemented with 10% FBS, 1 mM L-glutamine, and 100 μg/mL penicillin-streptomycin.

Hs68 cells and PBMCs were separately added to each well of 96-well plates and incubated for 24 h in a humidified incubator with an atmosphere of 95% air and 5% CO_2_ at 37 °C. Consequently, each *A. domesticus* extract (concentrations ranging from 3.125 to 100 µg/mL) was added and further incubated for 48 h. After incubation, the medium was discarded and MTT dye solution was added and maintained for 4 h. Subsequently, the supernatant was completely discarded. Then DMSO was added and mixed thoroughly to dissolve purple formazan crystals. The mixtures were measured for absorbance at 578 nm with a reference wavelength of 630 nm using an ELISA plate reader (Metertech Inc., Taipei, Taiwan). The percentage of cell viability was calculated using the following equation:

Cell viability (%) = [*A*/*B*] × 100, (2)

where *A* is the absorbance of the cells treated with each *A. domesticus* extract and *B* is the absorbance of the untreated cells. Each of the experiments was performed independently in triplicate.

### 3.8. Determination of TGF-β1-Stimulating Activities of A. domesticus Extracts

*A. domesticus* extracts were evaluated for TGF-β1 expression on Hs68 cells using enzyme-linked immunosorbent assay (ELISA) described by Lee et al. with slight adaptations [96]. Hs68 cells were seeded at a density of 1 × 10^4^ cells/mL with FBS-free DMEM and incubated for 24 h in a humidified incubator. After incubation, the medium was replaced with PBS, and the cells were then exposed to UVB irradiation (21.6 mJ/cm^2^) using UVB lamps, model number G9T5E (Sankyo Denki Co., Tokyo, Japan). The energy output of the UVB lamps was measured with a UVAB light meter, model number TM213 (Tenmars, Taipei, Taiwan). Subsequently, PBS was removed and each *A. domesticus* extract in FBS-free DMEM (concentration of 100 μg/mL) was added and further incubated for 72 h. The medium was collected for TGF-β1 level determination following the manufacturer’s protocol for Human TGF-beta 1 DuoSet ELISA (R&D System, Minneapolis, MN, USA). The results were reported as TGF-β1 expression (pg/mL). Non-irradiated cells were used as a control. Each of the experiments was performed independently in triplicate.

### 3.9. Determination of Anti-Skin-Aging Activities of A. domesticus Extracts

#### 3.9.1. Anti-Collagenase Activity Determination

The *A. domesticus* extracts were assessed for anti-collagenase activity using a spectrophotometric assay following Thring et al., with some modifications [97]. The results are presented as IC_50_ values representing the concentration of *A. domesticus* extracts suppressing collagenase activity by 50%. GraphPad/Prism program version 8.0 (GraphPad Software Inc., La Jolla, CA, USA) was used to plot the dose–response relationship and analyzed for IC_50_ values. Oleanolic acid, a triterpenoid compound widely revered and unequivocally recognized as a remarkable matrix metalloproteinase inhibitor [98,99,100], was used as a positive control. Each of the experiments was performed independently in triplicate.

#### 3.9.2. Anti-Hyaluronidase Activity Determination

The *A. domesticus* extracts were investigated for anti-hyaluronidase activity using a spectrophotometric assay adapted from Prommaban et al. [101]. The results were expressed as IC_50_ values representing the concentration of *A. domesticus* extracts suppressing hyaluronidase activity by 50%. GraphPad/Prism program version 8.0 (GraphPad Software Inc., La Jolla, CA, USA) was used to plot the dose–response relationship and analyzed for IC_50_ values. Oleanolic acid, a well-known acknowledged hyaluronidase inhibitor [102], was used as a positive control. Three experiments were performed independently.

### 3.10. Statistical Analysis

Where applicable, data are shown as mean  ±  standard deviation (S.D.) from three independently performed experiments. Statistical analysis was assessed with a one-way analysis of variance (ANOVA) followed by a post hoc Tukey test using GraphPad Prism version 8.0 (GraphPad Software Inc., La Jolla, CA, USA). A statistically significant difference was marked when *p* < 0.05.

## 4. Conclusions

This study was the first to investigate the chemical constituents, safety, and biological activities concerning the skin aging retardation of *A. domesticus* extracts. Each *A. domesticus* extract had its own unique appearance and odor. These extracts were found to encompass a range of chemical constituents, with AQ displaying notably higher total protein and phenolic content compared to all other extracts. Notably, AQ exhibited exceptional anti-skin-aging properties, evidenced by its potent TGF-β1 upregulation and MMP-1 suppression activities. All *A. domesticus* extracts were proposed as safe for topical application, since they induced no signs of irritation on the CAM and showed no cytotoxicity toward Hs68 cells or PBMCs. It is worth noting that the observed activities may not be solely attributed to specific compounds, as natural extracts comprise complex mixtures of diverse chemical molecules that interact synergistically or complementarily to contribute to their biological effects. Nonetheless, it is recommended that the AQ extract obtained from *A. domesticus*, which emerges as an appealing natural resource due to its blend of economic viability, operational simplicity, and cost-effectiveness, be considered for further development as an anti-skin-aging product in the cosmetic or cosmeceutical industries.

## Figures and Tables

**Table 1:** Chemical compositions of *A. domesticus* extracts (*n* = 3).

Extracts	Total Protein Content (% *w*/*w* Dry Weight Basis)	TPC (mg GAE/g Extract)	TFC (mg QE/g Extract)
AQ	63 ± 1 ^a^	0.48 ± 0.03 ^a^	0.02 ± 0.10 ^c^
ED	44 ± 4 ^b^	0.34 ± 0.03 ^b^	0.26 ± 0.02 ^a,b^
EM	34 ± 2 ^c^	0.26 ± 0.01 ^c^	0.33 ± 0.02 ^a^
HX	14 ± 1 ^d^	0.05 ± 0.03 ^d^	0.09 ± 0.08 ^b,c^

NOTE: TPC = total phenolic content; TFC = total flavonoid content; AQ = aqueous extract from thermal solvent extraction; ED = ethanolic extract from thermal solvent extraction; EM = ethanolic extract from maceration; HX = hexane extract from maceration. The data are shown as mean ± SD (*n* = 3). The lower-case letters (a, b, c, and d) indicate significant variability among *A. domesticus* extracts. Statistical significance was analyzed using one-way ANOVA followed by a post hoc Tukey test (*p* < 0.05).

**Table 2:** Protein molecular weight distribution of *A. domesticus* aqueous extract and its possible protein types.

Protein Band	MW (kDa)	Possible Protein Types
I	>260	Protein glycation, cross-linking, or aggregation [58]
II	~100	Sarcoplasmic/Endoplasmic reticulum calcium ATPase [57,58], alpha-actinin [58]
III	60	β-Glycosidase [55]
IV	50	Tubulin [57,58], troponin T [58]
V	40	Actin [42,57,58], monomeric arginine kinase [42,58]
VI	33	Tropomyosin [57,58]
VII	10–25	Cuticle proteins [42,55], proteolytic degradation [42]

NOTE: MW = molecular weight.

**Table 3:** Anti-skin-aging activities of *A. domesticus* extracts (*n* = 3).

Sample	IC_50_ (µg/mL)	
	Collagenase Inhibition	Hyaluronidase Inhibition
OA	3 ± 1 ^a^	29 ± 1 ^a^
AQ	26 ± 1 ^b^	181 ± 2 ^d^
ED	39 ± 2 ^c^	34 ± 2 ^a^
EM	61 ± 4 ^d^	47 ± 4 ^b^
HX	130 ± 3 ^e^	112 ± 5 ^c^

NOTE: IC_50_ = concentration of *A. domesticus* extracts suppressing collagenase or hyaluronidase activity by 50%; AQ = aqueous extract; ED = ethanolic extract from thermal solvent extraction; EM = ethanolic extract from maceration; HX = hexane extract; OA = oleanolic acid. Data are shown as mean ± SD (*n* = 3). Lower-case letters (a, b, c, d, and e) indicate significant variability among *A. domesticus* extracts. Statistical significance was analyzed using one-way ANOVA followed by post hoc Tukey test (*p* < 0.05).

## Data Availability

Data will be made available on request.
